# Diabetes knowledge and behaviour: a cross-sectional study of Jordanian adults

**DOI:** 10.1007/s00125-024-06304-3

**Published:** 2024-11-07

**Authors:** Rula A. Amr, Ahmed M. Al-Smadi, Rand T. Akasheh

**Affiliations:** 1 Department of Nutrition and Dietetics, American University of Madaba, Madaba, Jordan; 2 Department of Adult Health Nursing, Al al-Bayt University, Mafraq, Jordan; 3 Department of Internal Medicine, The Ohio State University, Columbus, OH USA; 4 Department of Pediatrics, University of Illinois at Chicago, Chicago, IL USA

**Keywords:** Clinical factors, Demographic factors, Diabetes knowledge, Diabetes mellitus, Health behaviours

## Abstract

**Aims/hypothesis:**

Diabetes mellitus is a significant global health concern that is projected to affect 7.7% of the global population by 2030. Understanding factors that influence diabetes knowledge and management adherence is crucial for effective diabetes mellitus management and prevention. This study investigates the relationships between demographic and clinical factors and their impact on diabetes knowledge and behaviour, as well as the potential influence of diabetes knowledge on management behaviours.

**Methods:**

The study comprised a cross-sectional survey of 1050 adults, collecting data on age, sex, marital status, education, employment, hypertension, dyslipidaemia (any lipid imbalance, such as high cholesterol, high LDL-cholesterol or low HDL-cholesterol), smoking and diabetes status. Two multiple linear regression models were used to identify factors associated with diabetes knowledge and behaviour, and a simple linear regression model was used to assess the relationship between knowledge and behaviour.

**Results:**

Significant associations were found between diabetes knowledge and the following factors: age (44.32 ± 9.53 for ≥50 years vs 39.73 ± 9.95 for 18 to <25 years; *p*<0.0001), sex (49.00 ± 12.35 for women vs 45.09 ± 13.27 for men; *p*<0.0001), marital status (50.92 ± 11.69 for married vs 45.39 ± 13.10 for single; *p*<0.0001), smoking status (45.78 ± 13.22 for smokers vs 48.22 ± 12.15 for non-smokers; *p*=0.003), hypertension (46.46 ± 13.11 for present vs 47.31 ± 12.87 for absent; *p*=0.007) and diabetes status (69.49 ± 17.35 for present vs 62.76 ± 16.88 for absent; *p*<0.001). Behaviour scores correlated similarly with these factors except for diabetes and smoking status. The adjusted simple linear regression model revealed that diabetes knowledge was significantly associated with better management behaviours (coefficient=0.0794, *p*<0.001) after adjusting for demographic and clinical factors.

**Conclusions/interpretation:**

This study highlights the importance of demographic and clinical factors in the context of diabetes knowledge and behaviours, underscoring the need for targeted educational and preventive programmes to improve diabetes management, especially in vulnerable populations. Additionally, the strong association between diabetes knowledge and management behaviours supports a knowledge–attitude–behaviour (KAB) model of diabetes management.

**Graphical Abstract:**

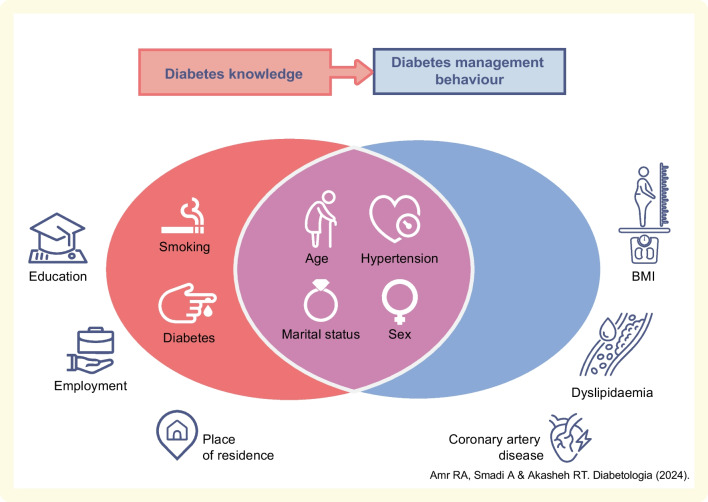


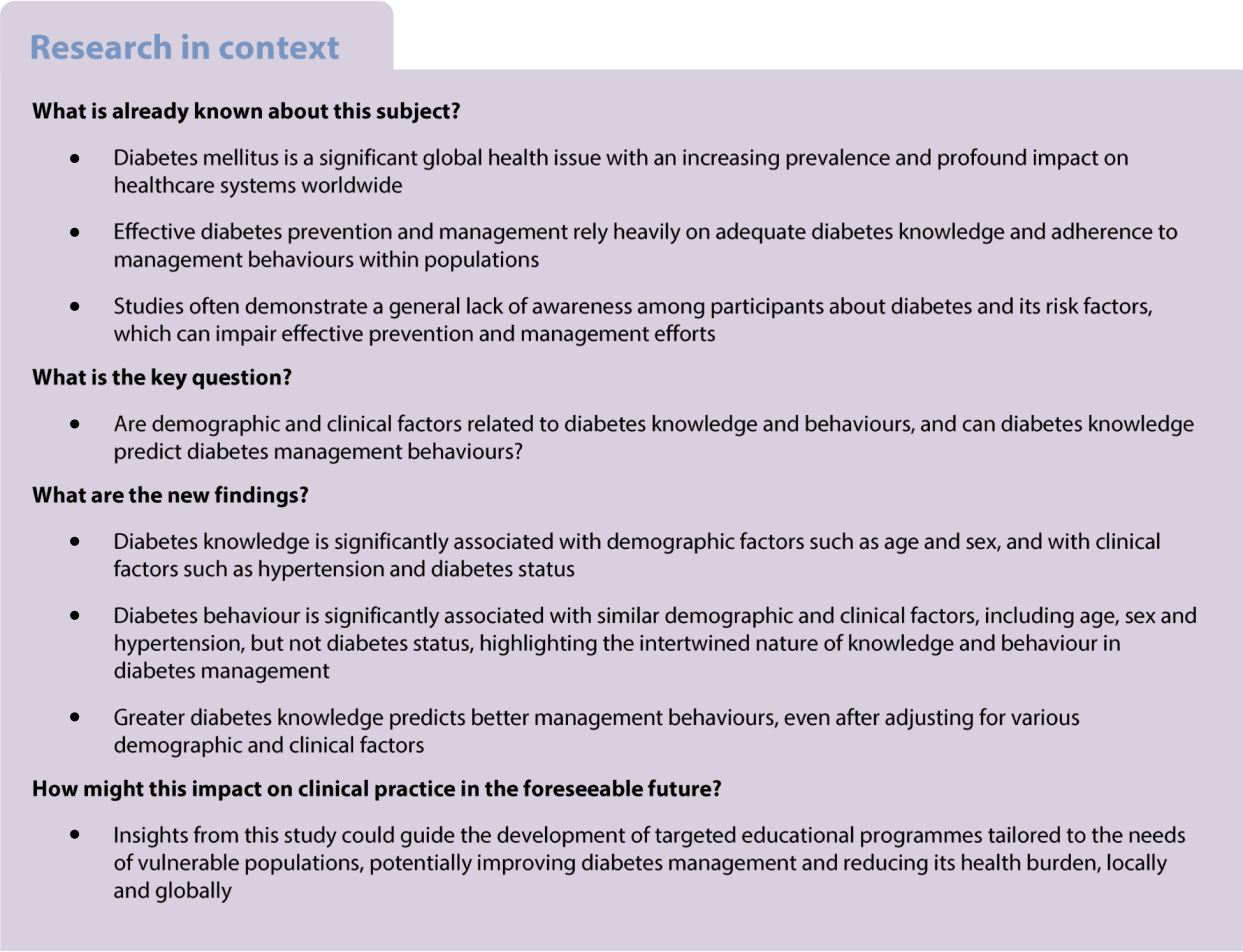


## Introduction

Diabetes mellitus is a widespread chronic illness with a projected global prevalence of 7.7% among adults by 2030 [1]. In Jordan, the diabetes mellitus prevalence reached 17.1% among adults in 2008 [2], and was estimated at approximately 10.3% in 2019 [3]. Diabetes can be distinguished by various classical symptoms, such as increased thirst and hunger and frequent urination, and has the potential to cause severe and enduring complications if not properly managed [4]. Prompt identification and management, including consistent physical activity, a nutritious diet, weight management and adherence to prescribed medications, can improve blood sugar regulation and reduce the likelihood of complications [4].

Comprehensive knowledge about diabetes mellitus and its complications is crucial for effective disease management. Patients with a strong understanding of diabetes mellitus and its potential complications are more likely to follow appropriate treatment and healthcare plans [5]. Surveys of knowledge, attitudes and practices are used to assess people’s understanding of and behaviours towards diabetes mellitus [6]. Numerous studies have highlighted the importance of raising awareness to manage risk factors and prevent diabetes mellitus [7–9]. It has been shown that educated people with diabetes tend to have better control of their condition, including non-pharmacological and pharmacological approaches [10, 11].

Many studies have investigated the extent of diabetes knowledge among people with diabetes. A sub-optimal level of knowledge was identified in several studies in many countries [12–18], and diabetes self-management and education have been shown to play a vital role in improving diabetes management [19].

Despite the high prevalence of diabetes in Jordan, the general population has limited knowledge of the disease and its risk factors. Previous studies have highlighted significant gaps in awareness, which hinder prevention, detection and management efforts, leading to adverse health outcomes and increased healthcare costs [20]. Therefore, improving knowledge about diabetes and its risk factors is critical.

This study aimed to investigate the relationships between demographic/clinical factors and diabetes knowledge/behaviour scores among Jordanian adults. The study also aimed to assess the relationship between diabetes knowledge and behaviour in the context of the knowledge–attitude–behaviour (KAB) model [16]. By identifying factors associated with diabetes knowledge and behaviours, and understanding how diabetes knowledge relates to adherence to management practices, this study provides crucial insights for developing targeted educational and preventive programmes to improve diabetes management and outcomes in Jordan.

## Methods

### Study design

The investigation relies on information gathered from a cross-sectional survey that aimed to examine the comprehension and behaviour concerning diabetes mellitus within a cohort of adults in Jordan.

### Sampling method

A convenience sampling technique was used to recruit participants for this cross-sectional study. Convenience sampling was chosen due to its practicality and cost-effectiveness, allowing for a broad and diverse range of participants to be included within a limited timeframe. This method facilitated the recruitment of participants from various easily accessible locations (such as healthcare centres, community centres, workplaces and public areas), ensuring a sufficiently large and varied sample to explore the relationships between demographic/clinical factors and diabetes knowledge/behaviour.

The sample size was determined through a power analysis to ensure adequate statistical power. Based on an estimated population of 1 million individuals, a minimum sample size of 1050 was calculated to achieve a 95% confidence level and a 3% margin of error. The sample size also accounted for expected prevalence rates of diabetes knowledge and behaviour to enable detection of meaningful differences across demographic and clinical groups. To recruit a representative sample, the study recruited 1050 female and male respondents, aged 18 to 84 years, from the cities of Amman, Madaba and Al-Karak, and with diverse socioeconomic backgrounds. No participant dropouts were recorded.

Individuals with cognitive impairments or language barriers that would prevent them from completing the survey were excluded.

### Ethical considerations

The investigation was performed in accordance with the Declaration of Helsinki, and the ethical committee of the American University of Madaba sanctioned all procedures performed in the participants (ethical approval number H23005). Written informed consent was obtained from all study participants.

### Study setting and participants

The study included 1050 Jordanian adults aged 18–84 years from various regions of Jordan, ensuring representation from both urban areas (*n*=921) and rural areas (*n*=129). Urban areas have a high population density and well-developed infrastructure, while rural areas have lower population density and less developed infrastructure. This distribution aimed to capture diverse demographic and clinical profiles, considering variations in healthcare access, socioeconomic status and lifestyle factors. The study was conducted over six months, from February 2023 to June 2023.

### Clinical characteristics

Participants’ clinical characteristics were classified based on self-reported medical history. Among the participants, 52 reported having diabetes and 998 reported not having diabetes. Additionally, 16 participants reported having dyslipidaemia (any lipid imbalance, such as high cholesterol, high LDL-cholesterol or low HDL-cholesterol), while 1034 did not. Furthermore, 11 participants reported having coronary artery disease, while 1039 did not.

### Data collection

Trained nutrition postgraduate researchers conducted data collection for this study. They briefed participants on the study’s objectives and methodologies, and ensured that informed consent was obtained prior to participation. The researchers assisted individuals with reading difficulties, ensuring accurate and complete responses. Participants completed the structured, self-administered questionnaire in approximately 35–40 minutes, and all responses were collected with strict adherence to confidentiality protocols. The data collection process aimed to maximise response accuracy and quality, providing participants with a supportive and secure environment throughout.

### Anthropometric assessment

#### Height

Trained senior undergraduate nutrition students gauged each participant’s height once using a measuring rod. Participants were instructed to stand with their feet together and knees extended, while positioning their heels, buttocks and shoulder blades against the wall. In addition, they were advised to align their head with the Frankfurt horizontal plane, as described by Nieman and Lee [21].

#### Body weight

A scale with a capacity of 150 kg and a precision of 0.1 kg was used to measure the participants’ body weights. Prior to each measurement, the scale was positioned on a flat, firm surface and calibrated to zero. Participants were requested to stand unaided on the central area of the platform with minimal attire and without footwear, facing straight ahead, as described by Nieman and Lee [21].

#### BMI

Body weight in kilograms divided by height in metres squared was used to calculate BMI. Subsequently, participant BMI values were classified into four categories: underweight (<18.5 kg/m^2^), normal weight (18.5–24.9 kg/m^2^), overweight (25–29.9 kg/m^2^) and obese (≥30 kg/m^2^), using the categorisation approach described by Nieman and Lee [21].

### Physical activity assessment

Participants’ weekly physical activity levels were categorised based on the 2005 International Physical Activity Questionnaire guidelines [22]. High-level physical activity was characterised by engaging in vigorous exercise for more than 3 days per week, accumulating 1500 metabolic equivalents (METs) or exceeding 3000 METs through a combination of walking or moderate to vigorous intensity activities over 7 days. Moderate-level physical activity was defined as performing vigorous exercise for over 20 min on more than 3 days per week, or engaging in moderate intensity exercise or walking for over 30 min on more than 5 days per week, or reaching 600 METs through walking or moderate to vigorous activities. Low-level physical activity was classified as not meeting the criteria for either high or moderate activity levels.

### Survey

To gather data for this research, a self-administered survey was conducted, using closed-ended questions. The survey covered various demographic and clinical details, including age, sex, education level, employment status, marital status, chronic illness, height and weight. It also included questions on behaviour and knowledge relating to diabetes mellitus, such as its symptoms, risk factors and complications. Data on national origin (Jordanian or non-Jordanian) were also collected. Race and ethnicity have not been well studied and identified in Jordan, as is the case in other Middle Eastern countries. Generally, people tend to identify themselves based on national origin [23], even though people with different national origins in the Middle East may share the same race/ethnicity. All the study participants were Jordanian.

The questionnaire was created and piloted in a previous study by Khan et al [24] based on literature reviews and the findings of previous studies [25–27]. They evaluated the validity of the questionnaire’s content through a review process involving professors with expertise in the research subject and a socio-psychologist [24]. The panel assessed the statements in the questionnaire to ensure that they adequately addressed the study’s objectives [24]. Permission to use the questionnaire for the current research was obtained from the authors.

### Survey validation

We performed rigorous validation of the survey tool through a series of detailed statistical analyses to establish the reliability and validity of the findings and to ensure that the tool is reliable in the Jordanian context. Initial data screening was performed to eliminate inconsistencies and missing values, ensuring that the dataset was clean and suitable for analysis.

First, principal component analysis was used to identify latent factors and uncover the underlying structure within the data [28]. The results of the principal component analysis showed a well-defined factor structure, with the explained variance for the first seven components as follows: component 1 (general knowledge on diabetes mellitus) accounted for 25.10% of the variance, component 2 (knowledge of risk factors) accounted for 17.30%, component 3 (knowledge of symptoms) accounted for 13.45%, component 4 (knowledge of complications) accounted for 12.20%, component 5 (health behaviour and medical history) accounted for 9.05%, component 6 (source of information on diabetes) accounted for 7.75%, and component 7 (perceived beneficial behavioural changes) accounted for 6.15%. These components collectively captured 91.00% of the total variance, indicating that the primary factors contributing to diabetes knowledge and behaviour were comprehensively represented.

The Kaiser–Meyer–Olkin measure was calculated to assess sampling adequacy for factor analysis [29]. The overall value was 0.81, which is considered excellent, indicating that the sample was adequate for principal component analysis and that the data were suitable for factor analysis. Bartlett’s test of sphericity was conducted to test whether the correlation matrix was an identity matrix [30], which would indicate that the variables were unrelated. The test showed a highly significant result, with a *χ*^2^ value of 2580.45 (*p*<0.001), confirming that the correlations between items were sufficiently large for principal component analysis, thereby validating the factorability of the correlation matrix.

Cronbach’s alpha was calculated to ensure internal consistency. The results showed strong internal consistency for the primary factors, with a Cronbach’s alpha value of 0.815, indicating that the items within each factor reliably measured the same underlying construct [31]. This high level of internal consistency provides confidence in the reliability of the survey tool.

Additionally, ridge regression was applied and achieved a cross-validation mean score of 0.987 with a low SD (0.025) and a model score of 1.0, indicating very high goodness of fit. This confirms that the model effectively captured the relationship between the variables, further validating the reliability of the survey tool. Principal component regression was also employed, achieving a cross-validation mean score of 0.986 with a low SD (0.027) and a model score of 0.9992. This indicates a robust fit without overfitting, further supporting the validity of the survey tool [32].

### Data scoring and aggregation

For the knowledge component, participants were asked a series of questions aimed at assessing their understanding of diabetes, covering symptoms, risk factors, complications and general knowledge. Responses were scored on a binary scale, with ‘1’ assigned for correct answers and ‘0’ for incorrect answers. The sum of these binary scores provided a total knowledge score for each participant, which was then normalised to the total number of knowledge-related questions to derive a percentage score. Similarly, the behaviour component, evaluating adherence to recommended diabetes management practices such as medication, dietary practices and physical activity levels, was scored. Each behaviour that adhered to guidelines was scored as ‘1’, and non-adherent behaviours were scored as ‘0’. A total behaviour score was calculated for each participant by summing these scores and normalising them to the total number of behaviour-related questions to derive a percentage score.

### Statistical analysis

The numbers of participants in the various demographic and clinical categories were determined and percentages were calculated to provide clarity on group distribution. Scores for knowledge and behaviour were aggregated by demographic profiles (e.g. age/sex) and clinical profiles (e.g. hypertension/BMI), with mean scores and SDs indicating central tendency and dispersion.

Differences in knowledge and behaviour scores across categories were tested using unpaired *t* test for binary factors (e.g. sex or marital status) and one-way ANOVA for multi-level factors (e.g. age or BMI), with statistical significance set at *p*<0.05.

Two multiple linear regression models were developed to explore the relationship between various demographic/clinical variables and diabetes knowledge/behaviours. A simple linear regression model was developed to test the association between diabetes knowledge and behaviours. Variables that demonstrated significant associations (*p*<0.05) in preliminary *t* tests and ANOVA were included in the regression models. Additionally, covariate adjustment was guided by previous research showing significant associations between diabetes knowledge/behaviours and factors such as age [33], sex [34], marital status [35], hypertension [36] and smoking status [37].

Diagnostic tests were performed to ensure the validity of the regression analysis, including checks for linearity, multicollinearity (variance inflation factor), homoscedasticity, and normality of residuals (Shapiro–Wilk and Durbin–Watson). The models quantified the influence of each factor, calculating coefficients, standard errors and *R*^2^ values, thereby explaining the proportion of variance in diabetes knowledge and behaviour scores attributable to the included variables. Data analysis was conducted using SPSS version 28 (IBM, USA).

## Results

The study included 1050 Jordanian participants, aged 18 to <25 years (51.62%), 25 to <50 years (36.38%) or ≥50 years (12.00%). They were predominantly female (54.86%), single (62.10%), educated beyond secondary level (83.24%) and urban residents (87.71%). Moreover, 43.05% of the participants were smokers and 81.05% reported low physical activity. Additionally, 50.95% had normal BMI, 32.86% had overweight and 16.19% had obesity. The prevalence rates of hypertension, dyslipidaemia and coronary artery disease were 6.86%, 1.52% and 1.05%, respectively, while diabetes affected 4.95% of the participants (Table 1).

**Table 1:** Distribution of diabetes knowledge and behaviour scores across demographic and clinical categories

Variable	*n* (%)	Diabetes knowledge score	*p* value	Diabetes behaviour score	*p* value
Age (years)					
18 to <25	542 (51.62)	39.73 ± 9.95	<0.0001	82.42 ± 21.84	<0.0001
25 to <50	382 (36.38)	42.61 ± 9.18		88.06 ± 17.72	
50 and above	126 (12.00)	44.32 ± 9.53		88.57 ± 18.27	
Sex					
Male	474 (45.14)	45.09 ± 13.27	<0.0001	81.81 ± 22.00	<0.0001
Female	576 (54.86)	49.00 ± 12.35		88.00 ± 18.14	
Marital status					
Single	652 (62.10)	45.39 ± 13.10	<0.0001	83.47 ± 20.91	0.0003
Married	398 (37.90)	50.92 ± 11.69		88.53 ± 18.11	
Education					
Secondary or less	176 (16.76)	47.04 ± 14.58	0.457	85.06 ± 21.33	0.913
Higher than secondary	874 (83.24)	47.27 ± 12.56		85.24 ± 19.98	
Employment					
Employed	431 (41.05)	47.70 ± 12.35	0.745	85.80 ± 20.42	0.429
Unemployed	619 (58.95)	46.91 ± 13.29		84.80 ± 20.05	
Hypertension					
Yes	72 (6.86)	46.46 ± 13.11	0.007	84.46 ± 21.75	0.017
No	978 (93.14)	47.31 ± 12.87		85.10 ± 19.90	
Dyslipidaemia					
Yes	16 (1.52)	47.12 ± 11.34	0.076	86.25 ± 22.45	0.228
No	1034 (98.48)	47.25 ± 12.98		85.00 ± 19.98	
Coronary artery disease					
Yes	11 (1.05)	47.45 ± 10.58	0.975	82.73 ± 20.58	0.849
No	1039 (98.95)	47.19 ± 12.77		85.20 ± 20.00	
Smoking					
Yes	452 (43.05)	45.78 ± 13.22	0.003	84.09 ± 21.32	0.076
No	598 (56.95)	48.22 ± 12.15		86.15 ± 19.43	
Physical activity					
Low	851 (81.05)	46.37 ± 12.73	0.200	83.40 ± 20.56	0.507
Moderate	91 (8.67)	47.90 ± 11.88		87.77 ± 18.22	
High	108 (10.29)	48.35 ± 13.07		90.08 ± 17.95	
BMI^a^					
Normal	535 (50.95)	46.52 ± 12.99	0.089	84.52 ± 20.12	0.108
Overweight	345 (32.86)	48.60 ± 11.82		86.70 ± 18.98	
Obesity	170 (16.19)	47.13 ± 12.20		83.05 ± 21.64	
Diabetes status					
Yes	52 (4.95)	69.49 ± 17.35	<0.001	88.08 ± 20.39	0.294
No	998 (95.05)	62.76 ± 16.88		85.06 ± 20.19	
Location					
Urban	921 (87.71)	75.60 ± 20.90	0.1108	84.99 ± 20.29	0.3571
Rural	129 (12.29)	78.71 ± 19.35		86.74 ± 19.53	

Data for the diabetes knowledge and behaviour scores are presented as means ± SD. The score represents the percentage of correct responses to questions on diabetes knowledge and behaviours

^a^BMI values were classified as underweight (<18.5 kg/m^2^ [although there were no participants in this category]), normal weight (18.5–24.9 kg/m^2^), overweight (25–29.9 kg/m^2^) and obese (≥30 kg/m^2^)

A *p* value <0.05 indicates statistically significant differences between categories within a variable, based on Student’s *t* tests for variables with two categories and one-way ANOVA for variables with three or more categories

Diabetes knowledge and behaviour scores were calculated across various demographic and clinical categories, and significant associations were observed between certain factors and these scores (Table 1). Older participants scored significantly higher for knowledge, with the group aged ≥50 years having a significantly higher mean score (44.32 ± 9.53) compared with the youngest group (aged 18 to <25 years), who had a mean score of (39.73 ± 9.95, *p*<0.0001). The behaviour scores were also higher in the older age groups, with the group aged ≥50 years scoring 88.57 ± 18.27, compared with 82.42 ± 21.84 in the youngest group (*p*<0.0001). Women demonstrated higher knowledge scores (49.00 ± 12.35) than men (45.09 ± 13.27, *p*<0.0001). Behaviour scores were also higher for women (88.00 ± 18.14) than men (81.81 ± 22.00, *p*<0.0001). Additionally, married participants had higher knowledge scores (50.92 ± 11.69) than single participants (45.39 ± 13.10, *p*<0.0001). Behaviour scores were also higher among married participants (88.53 ± 18.11) compared with single participants (83.47 ± 20.91, *p*=0.0003). Non-hypertensive individuals had higher knowledge scores (47.31 ± 12.87) than those with hypertension (46.46 ± 13.11, *p*=0.007). Behaviour scores were also higher in non-hypertensive individuals (85.10 ± 19.90) compared with hypertensive individuals (84.46 ± 21.75, *p*=0.017). Participants with diabetes had higher knowledge scores (69.49 ± 17.35) than those without diabetes (62.76 ± 16.88, *p*<0.001), and non-smokers had higher knowledge scores (48.22 ± 12.15) than smokers (45.78 ± 13.22, *p*=0.003) (Table 1).

The multiple linear regression analysis identified significant associations between clinical/demographic factors and adjusted diabetes knowledge scores (Table 2). The model was statistically significant (*F* statistic=31.67, *p*<0.0001), and explained 12.8% of the variance in diabetes knowledge scores (adjusted *R*^2^=0.128). Sex (female vs male) had a significant positive coefficient (2.1991, *p*<0.0001), indicating higher adjusted knowledge scores among women. Hypertension status had a significant positive coefficient (4.5346, *p*<0.0001), indicating higher adjusted knowledge scores among individuals with hypertension. Diabetes status (people with diabetes vs those without diabetes) also showed a significant positive coefficient (3.1125, *p*<0.0001), suggesting higher adjusted knowledge scores among individuals with diabetes. Age was non-significantly but positively associated with adjusted diabetes knowledge scores (*p*=0.068), while marital and smoking status were not significantly associated with adjusted diabetes knowledge scores (Table 2).

**Table 2:** Multiple linear regression analysis for predictors of diabetes knowledge

Variable	Coefficient	SE	*p* value
Intercept (constant)	−4.68 × 10^13^	4.61 × 10^13^	0.311
Age	0.9827	0.538	0.068
Sex (female)	2.1991	0.515	<0.0001
Marital status	0.4359	0.764	0.568
Hypertension	4.5346	0.558	<0.0001
Diabetes status	3.1125	0.568	<0.0001
Smoking status	0.6745	0.5643	0.232

Data are presented as coefficient ± SE, where the coefficient represents the estimated change in the dependent variable for each unit increase in the predictor variable, assuming other variables are held constant

A *p* value <0.05 indicates statistical significance

The multiple linear regression analysis also identified significant associations between clinical/demographic factors and adjusted diabetes behaviour scores (Table 3). The model explained 10.9% of the variance in diabetes behaviour scores (adjusted *R*^2^=0.109), and was statistically significant (*F* statistic=33.24, *p*<0.0001). The intercept coefficient was significant (3.3326, *p*<0.0001), indicating the baseline score for diabetes behaviour when all predictor variables are zero. Age showed a significant positive coefficient (0.3284, *p*=0.010), indicating that older age is associated with higher adjusted diabetes behaviour scores. Sex (female vs male) had a significant positive coefficient (0.5914, *p*<0.0001), suggesting higher adjusted behaviour scores among women. Furthermore, hypertension status had a significant positive coefficient (0.5410, *p*<0.0001), indicating higher adjusted behaviour scores among individuals with hypertension. Marital and smoking status were not significantly associated with adjusted diabetes behaviour scores (Table 3).

**Table 3:** Multiple linear regression analysis for predictors of diabetes behaviour

Variable	Coefficient	SE	*p* value
Intercept (constant)	3.3326	0.133	<0.0001
Age	0.3284	0.127	0.010
Sex (female)	0.5914	0.123	<0.0001
Marital status	0.0246	0.182	0.893
Hypertension	0.5410	0.133	<0.0001
Smoking status	−0.0019	0.1301	0.988

Data are presented as coefficient ± SE, where the coefficient represents the estimated change in the dependent variable for each unit increase in the predictor variable, assuming other variables are held constant

A *p* value <0.05 indicates statistical significance

**Fig. 1:**
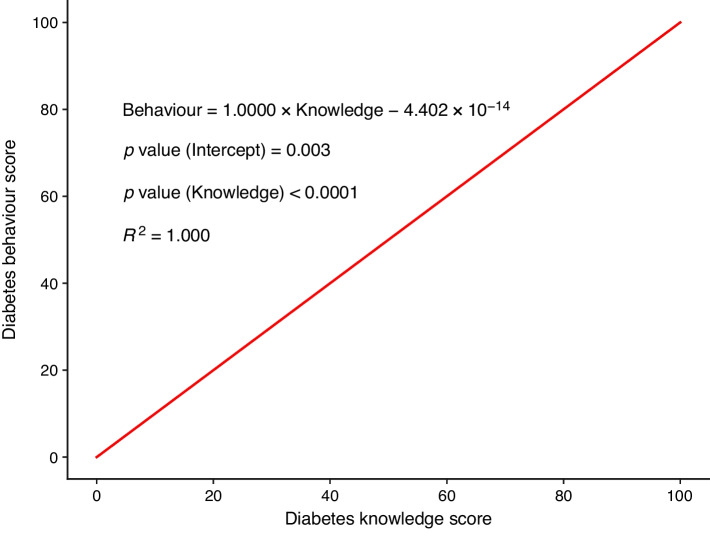
Diabetes knowledge is positively associated with diabetes behaviour, based on a simple linear regression analysis. The regression equation is formulated as: dependent variable = coefficient × independent variable – intercept. The coefficient represents the expected change in the dependent variable (behaviour score) for each unit increase in the independent variable (knowledge score). The *R*^2^ value of 1.000 indicates a strong positive association and a perfect fit of the model. A *p* value <0.05 indicates statistical significance

The simple linear regression analysis identified a significant association between diabetes knowledge and behaviour scores (Fig. 1). The model achieved an *R*^2^ value of 1.000, indicating a perfect fit. The intercept coefficient was significant (−4.402 × 10^−14^, *p*=0.003). The total knowledge score showed a positive coefficient (1.0000, *p*<0.0001), indicating a direct relationship between higher diabetes knowledge scores and higher diabetes behaviour scores. The regression model was then adjusted for age, sex, marital status, hypertension and smoking status. The positive association between diabetes knowledge scores and behaviour scores remained significant after adjustments, with a positive coefficient (0.0794, *p*<0.001) and significant intercept (4.9437, *p*<0.001). This confirms that higher diabetes knowledge scores are associated with better diabetes behaviour scores.

## Discussion

This study explored the impact of demographic and clinical factors on diabetes knowledge and behaviour scores among Jordanian adults. Older adults showed higher scores, suggesting increased health literacy, possibly resulting from frequent healthcare interactions and chronic condition management, in agreement with previous findings [33]. However, another study found no significant link between age and diabetes knowledge [38]. To our knowledge, the present study is unique in quantifying the impact of age in a Middle Eastern context, offering new insights into diabetes management within this population. These findings underscore the importance of culturally appropriate educational initiatives to bridge knowledge gaps.

Our study is unique to the Jordanian context and provided new insights into the influence of sex on diabetes knowledge and management in a Middle Eastern country. Women demonstrated significantly higher diabetes knowledge and behaviour scores than men, possibly due to proactive health management and frequent interactions with healthcare providers. This aligns with studies that have suggested that women’s caregiving roles contribute to higher health literacy [39, 40]. Another study on a sample of Portuguese adults found that women scored better than men in some aspects of diabetes knowledge [34]. However, it is noteworthy that sex disparities diminish with better healthcare, education access and sex equality [41]. Thus, addressing social support, healthcare access and educational opportunities is essential to equalise diabetes knowledge and behaviours between sexes.

Participants with hypertension exhibited higher diabetes knowledge and behaviour scores, possibly reflecting a broader understanding of chronic disease management. As reported by a previous study, the overlap in dietary and lifestyle management strategies for hypertension and diabetes may enhance overall diabetes comprehension among individuals with hypertension [42]. Thus, regular physical activity, a balanced diet and the frequent healthcare interactions inherent in hypertension management may contribute to better diabetes practices. Additionally, individuals with hypertension often receive education on both conditions, enhancing their health literacy. Patients who are managing multiple chronic conditions typically gain extensive health knowledge, becoming more informed and proactive in their care [36]. The results of our study suggest that the integration of treatment strategies across health domains in Jordan enables better diabetes knowledge and behaviour.

Individuals with diabetes showed significantly higher knowledge scores than those without diabetes, in agreement with findings that frequent disease management boosts diabetes understanding [43]. However, this did not translate to better behaviour scores, indicating barriers to effective management. Increased diabetes knowledge may not always lead to improved management behaviours due to insufficient health literacy and difficulty applying this knowledge in daily life. For example, socioeconomic constraints limit access to healthcare, medications and nutritious food, which are essential for proper diabetes management [44]. Access to local health services and educational programmes also affects health behaviours, particularly in under-served regions [45]. The findings of the current study suggest that barriers that impede the implementation of diabetes knowledge may exist within the Jordanian context. Therefore, future studies should investigate the attitudes and potential barriers that prevent the translation of knowledge into good diabetes management behaviours.

Diabetes knowledge is crucial for effective diabetes management. Our analysis, which is supported by the KAB model [16], revealed a strong relationship between diabetes knowledge and management behaviours, suggesting that greater knowledge is a precursor to better adherence to recommended practices. However, this was not true for participants with diabetes, who exhibited higher diabetes knowledge, but not behaviour, compared to participants without diabetes. Importantly, the KAB model suggests that behavioural change can be achieved by targeting both knowledge and attitudes, which entails provision of accurate information and promotion of positive attitudes [16]. Thus, while knowledge is essential, effective diabetes management must change attitudes and address barriers to ensure behavioural change.

The novelty of this study lies in its focus on the Jordanian context, highlighting how local demographic and clinical factors may influence the translation of knowledge into effective diabetes management behaviours, and stressing the importance of tailoring educational interventions to achieve desired behavioural changes. Additionally, while this study suggests that knowledge is a precursor to behaviour, supporting use of the KAB model as a framework for diabetes management, evaluating and modifying attitudes towards diabetes management recommendations and eliminating barriers remain essential.

### Study limitations

This study has several limitations. The cross-sectional design captures only a single-time relationship snapshot, preventing causal conclusions. The use of self-reported data may introduce biases such as social desirability bias and recall bias, while the use of convenience sampling means that the sample may not fully represent the entire population, especially those from remote or minority areas. Additionally, cultural and healthcare differences between Jordan and other regions caution against broad generalisation of the findings. Moreover, biochemical, psychosocial and ethnic/racial factors were not included in the analysis. Finally, patients with type 1 diabetes score better than those with type 2 diabetes in diabetes knowledge tests [46]. However, our study did not distinguish participants by diabetes type, thus disregarding the challenges that are specific to each type. Future studies should consider this distinction to better tailor interventions and educational programmes to the specific needs of type 1 and type 2 diabetes patients.

### Conclusion and implications

Diabetes knowledge is significantly associated with diabetes management behaviours in a sample of Jordanian adults, supporting the use of a KAB model for diabetes education. However, the association depends on several demographic and clinical factors, including age, sex and disease status. This highlights key implications for diabetes education and management in Jordan, suggesting the need for educational programmes tailored to people with specific demographic and clinical features. Future studies may investigate whether integrating education within primary care could increase diabetes knowledge and improve management behaviours and potentially health outcomes. Furthermore, evaluating attitudes and barriers that stand against adherence to diabetes management guidelines among vulnerable populations remains crucial. These findings advocate for a holistic approach to diabetes management in Jordan that utilises demographic insights for more effective interventions.

## Data Availability

Data supporting the results reported in this paper are available upon request from the corresponding author.

## Abbreviations

KAB: Knowledge–attitude–behaviour
METs: Metabolic equivalents

## References

[1] Shaw JE, Sicree RA, Zimmet PZ (2010) Global estimates of the prevalence of diabetes for 2010 and 2030. Diabetes Res Clin Pract 87(1):4–14. 10.1016/j.diabres.2009.10.007 19896746 10.1016/j.diabres.2009.10.007

[2] Ajlouni K, Khader YS, Batieha A, Ajlouni H, El-Khateeb M (2008) An increase in prevalence of diabetes mellitus in Jordan over 10 years. J Diabetes Complications 22(5):317–324. 10.1016/j.jdiacomp.2007.01.004 18413210 10.1016/j.jdiacomp.2007.01.004

[3] Haddad JA, Al Hyari MA, Al Momani MS, Al Omari AA, Ammari FL, Annabi FO (2020) Baseline characteristics and treatment pattern of type 2 diabetes patients in Jordan: analysis from the DISCOVER patient population. Alexandria J Med 56(1):51–55. 10.1080/20905068.2020.1747733

[4] Nathan DM, Buse JB, Davidson MB et al (2009) Medical management of hyperglycemia in type 2 diabetes: a consensus algorithm for the initiation and adjustment of therapy: a consensus statement of the American Diabetes Association and the European Association for the Study of Diabetes. Diabetes Care 32(1):193–203. 10.2337/dc08-9025 18945920 10.2337/dc08-9025 PMC2606813

[5] Shrivastava SR, Shrivastava PS, Ramasamy J (2013) Role of self-care in management of diabetes mellitus. J Diabetes Metab Dis 12(1):1–5 10.1186/2251-6581-12-14 PMC3599009 23497559

[6] Médicins du Monde (2011) The KAP survey model (knowledge, attitudes, & practices). US Agency for International Development, Washington DC

[7] Islam FMA, Chakrabarti R, Dirani M et al (2014) Knowledge, attitudes and practice of diabetes in rural Bangladesh: the Bangladesh Population based Diabetes and Eye Study (BPDES). PLoS One 9(10):e110368 25313643 10.1371/journal.pone.0110368 PMC4196995

[8] Deepa M, Deepa R, Shanthirani C et al (2005) Awareness and knowledge of diabetes in Chennai – the Chennai urban rural epidemiology study [CURES-9]. J Assoc Physicians India 53(4):283–287 15987011

[9] Shah VN, Kamdar P, Shah N (2009) Assessing the knowledge, attitudes and practice of type 2 diabetes among patients of Saurashtra region, Gujarat. Int J Diabetes Dev Ctries 29(3):118. 10.4103/0973-3930.54288 20165648 10.4103/0973-3930.54288 PMC2822215

[10] Powers MA, Bardsley J, Cypress M et al (2015) Diabetes self-management education and support in type 2 diabetes: a joint position statement of the American Diabetes Association, the American Association of Diabetes Educators, and the Academy of Nutrition and Dietetics. The Diabetes Educator 41(4):417–430. 10.1177/0145721715588904 26047627 10.1177/0145721715588904

[11] Rani PK, Raman R, Subramani S, Perumal G, Kumaramanickavel G, Sharma T (2008) Knowledge of diabetes and diabetic retinopathy among rural populations in India, and the influence of knowledge of diabetic retinopathy on attitude and practice. Rural Remote Health 8(3):1–9 18656993

[12] Angeles-Llerenas A, Carbajal-Sánchez N, Allen B, Zamora-Muñoz S, Lazcano-Ponce E (2005) Gender, body mass index and socio-demographic variables associated with knowledge about type 2 diabetes mellitus among 13 293 Mexican students. Acta Diabetol 42:36–45. 10.1007/s00592-005-0172-4 15868112 10.1007/s00592-005-0172-4

[13] Al Shafaee MA, Al-Shukaili S, Rizvi SGA et al (2008) Knowledge and perceptions of diabetes in a semi-urban Omani population. BMC Public Health 8:1–8 18644163 10.1186/1471-2458-8-249 PMC2517595

[14] Bruce DG, Davis WA, Cull CA, Davis TM (2003) Diabetes education and knowledge in patients with type 2 diabetes from the community: the Fremantle Diabetes Study. J Diabetes Complications 17(2):82–89. 10.1016/S1056-8727(02)00191-5 12614974 10.1016/s1056-8727(02)00191-5

[15] Gunay T, Ulusel B, Velipasaoglu S, Unal B, Ucku R, Ozgener N (2006) Factors affecting adult knowledge of diabetes in Narlidere Health District, Turkey. Acta Diabetol 43:142–147. 10.1007/s00592-006-0230-6 17211566 10.1007/s00592-006-0230-6

[16] Schrader PG, Lawless KA (2004) The knowledge, attitudes, & behaviors approach how to evaluate performance and learning in complex environments. Perform Improv 43(9):8–15. 10.1002/pfi.4140430905

[17] Murata G, Shah J, Adam K et al (2003) Factors affecting diabetes knowledge in type 2 diabetic veterans. Diabetologia 46:1170–1178. 10.1007/s00125-003-1161-1 12856126 10.1007/s00125-003-1161-1

[18] McClean M, McElnay J, Andrews W (2001) The association of psychosocial and diabetes factors to diabetes knowledge. Int J Pharmacy Practice 9(Suppl 1):9. 10.1111/j.2042-7174.2001.tb01069.x

[19] Funnell MM, Brown TL, Childs BP et al (2007) National standards for diabetes self-management education. The Diabetes Educator 33(4):599–614. 10.1177/0145721707305880 17684162 10.1177/0145721707305880

[20] Al-Latayfeh M, Shatnawi R, Al Shdaifat AA (2021) Attitudes and awareness towards diabetic retinopathy among patients with diabetes in Amman, Jordan. Diabetes Metab Syndr Obes 14:1425–1430. 10.2147/DMSO.S302554 33814919 10.2147/DMSO.S302554 PMC8009339

[21] Nieman DC, Lee R (2019) Nutritional assessment. McGraw-Hill Education, New York

[22] IPAQ Research Committee (2005) Guidelines for data processing and analysis of the International Physical Activity Questionnaire (IPAQ)-short and long forms

[23] Awad GA, Hashem H, Nguyen H (2021) Identity and ethnic/racial self-labeling among Americans of Arab or Middle Eastern and North African Descent. Identity 21(2):115–130. 10.1080/15283488.2021.1883277 38736970 10.1080/15283488.2021.1883277 PMC11086952

[24] Khan N, Gomathi KG, Shehnaz SI, Muttappallymyalil J (2012) Diabetes mellitus-related knowledge among university students in Ajman, United Arab Emirates. Sultan Qaboos University Med J 12(3):306. 10.12816/0003144 10.12816/0003144 PMC3413621 22912923

[25] Aljoudi AS, Taha AZ (2009) Knowledge of diabetes risk factors and preventive measures among attendees of a primary care center in eastern Saudi Arabia. Ann Saudi Med 29(1):15–19. 10.4103/0256-4947.51813 19139622 10.4103/0256-4947.51813 PMC2813608

[26] Mohieldein AH, Alzohairy MA, Hasan M (2011) Awareness of diabetes mellitus among Saudi non-diabetic population in Al-Qassim region, Saudi Arabia. J Diabetes Endocrinol 2(2):14–19

[27] Wee H, Ho H, Li S (2002) Public awareness of diabetes mellitus in Singapore. Singapore Med J 43(3):128–134 12005338

[28] Jolliffe IT (2002) Principal component analysis for special types of data. Springer, New York

[29] Kaiser HF (1974) An index of factorial simplicity. Psychometrika 39(1):31–36. 10.1007/BF02291575

[30] Shrestha N (2021) Factor analysis as a tool for survey analysis. Am J Appl Maths Stat 9(1):4–11. 10.12691/ajams-9-1-2

[31] Cronbach LJ (1951) Coefficient alpha and the internal structure of tests. Psychometrika 16(3):297–334. 10.1007/BF02310555

[32] Brown TA (2015) Confirmatory factor analysis for applied research. Guilford Press, New York

[33] Ong-Artborirak P, Seangpraw K, Boonyathee S, Auttama N, Winaiprasert P (2023) Health literacy, self-efficacy, self-care behaviors, and glycemic control among older adults with type 2 diabetes mellitus: a cross-sectional study in Thai communities. BMC Geriatrics 23(1):297. 10.1186/s12877-023-04010-0 37193967 10.1186/s12877-023-04010-0 PMC10185940

[34] Dos Santos PFL, Dos Santos PR, Ferrari GSL, Fonseca GAA, Ferrari CKB (2014) Knowledge of diabetes mellitus: does gender make a difference? Osong Public Health Res Perspect 5(4):199–203. 10.1016/j.phrp.2014.06.004 25379370 10.1016/j.phrp.2014.06.004 PMC4215000

[35] Ramezankhani A, Azizi F, Hadaegh F (2019) Associations of marital status with diabetes, hypertension, cardiovascular disease and all-cause mortality: a long term follow-up study. PLoS One 14(4):e0215593. 10.1371/journal.pone.0215593 31009512 10.1371/journal.pone.0215593 PMC6476533

[36] Singh S, Gupta NR, Raza ST, Kapoor A, Singh P (2021) Association of hypertension and its risk factor in type II diabetes mellitus patients. Asian J Med Sci 12(1):28–33. 10.3126/ajms.v12i1.30508

[37] Al-Adsani A, Moussa M, Al-Jasem L, Abdella N, Al-Hamad N (2009) The level and determinants of diabetes knowledge in Kuwaiti adults with type 2 diabetes. Diabetes Metab 35(2):121–128. 10.1016/j.diabet.2008.09.005 19250850 10.1016/j.diabet.2008.09.005

[38] Dzerounian J, Pirrie M, AlShenaiber L, Angeles R, Marzanek F, Agarwal G (2022) Health knowledge and self-efficacy to make health behaviour changes: a survey of older adults living in Ontario social housing. BMC Geriatrics 22(1):473. 10.1186/s12877-022-03116-1 35650537 10.1186/s12877-022-03116-1 PMC9158350

[39] Franconi F, Campesi I, Occhioni S, Tonolo G (2012) Sex-gender differences in diabetes vascular complications and treatment. Endocr Metab Immune Disord Drug Targets 12(2):179–196. 10.2174/187153012800493512 22236023 10.2174/187153012800493512

[40] Lundberg PC, Thrakul S (2012) Type 2 diabetes: how do Thai Buddhist people with diabetes practise self-management? J Adv Nurs 68(3):550–558. 10.1111/j.1365-2648.2011.05756.x 21711465 10.1111/j.1365-2648.2011.05756.x

[41] Yu MK, Lyles CR, Bent-Shaw LA, Young BA (2013) Sex disparities in diabetes process of care measures and self-care in high-risk patients. J Diabetes Res 2013:575814. 10.1155/2013/575814 23671877 10.1155/2013/575814 PMC3647593

[42] Mazzuchello FR, Tuon L, Simões PW et al (2016) Knowledge, attitudes and adherence to treatment in individuals with hypertension and diabetes mellitus. Mundo Saúde 40(4):418–432. 10.15343/0104-7809.20164004418432

[43] Zowgar AM, Siddiqui MI, Alattas KM (2018) Level of diabetes knowledge among adult patients with diabetes using diabetes knowledge test. Saudi Med J 39(2):161. 10.15537/smj.2017.2.21343 29436565 10.15537/smj.2017.2.21343 PMC5885093

[44] Stallwood L (2006) Relationship between caregiver knowledge and socioeconomic factors on glycemic outcomes of young children with diabetes. J Spec Pediatr Nurs 11(3):158–165. 10.1111/j.1744-6155.2006.00062.x 16774526 10.1111/j.1744-6155.2006.00062.x

[45] Correia JC, Lachat S, Lagger G et al (2019) Interventions targeting hypertension and diabetes mellitus at community and primary healthcare level in low-and middle-income countries: a scoping review. BMC Public Health 19:1–20 31752801 10.1186/s12889-019-7842-6 PMC6873661

[46] Fitzgerald JT, Funnell MM, Hess GE et al (1998) The reliability and validity of a brief diabetes knowledge test. Diabetes Care 21(5):706–710. 10.2337/diacare.21.5.706 9589228 10.2337/diacare.21.5.706

